# Efficacy and safety analysis of trastuzumab and paclitaxel based regimen plus carboplatin or epirubicin as neoadjuvant therapy for clinical stage II-III, HER2-positive breast cancer patients: a phase 2, open-label, multicenter, randomized trial

**DOI:** 10.18632/oncotarget.4337

**Published:** 2015-06-02

**Authors:** Liang Huang, Sheng Chen, Wentao Yang, Binghe Xu, Tao Huang, Hongjian Yang, Hong Zheng, Yongsheng Wang, Erwei Song, Jin Zhang, Shude Cui, Da Pang, Lili Tang, Yutao Lei, Cuizhi Geng, Zhiming Shao

**Affiliations:** ^1^ Department of Breast Surgery, Fudan University Shanghai Cancer Center/Cancer Institute, Shanghai, China; ^2^ Department of Oncology, Shanghai Medical College, Fudan University, Shanghai, China; ^3^ Department of Pathology, Cancer Center/Cancer Institute, Shanghai, China; ^4^ Department of Medical Oncology, Cancer Institute and Cancer Hospital, Chinese Academy of Medical Sciences and Peking Union Medical College, Beijing, China; ^5^ Department of Breast and Thyroid Surgery, Union Hospital, Tongji Medical College, Huazhong University of Science and Technology, Wuhan, China; ^6^ Department of Breast Tumor Surgery, Zhejiang Cancer Hospital, Hangzhou, Zhejiang, China; ^7^ Department of Head and Neck and Mammary Oncology, Cancer Center and State Key Laboratory of Biotherapy, Laboratory of Molecular Diagnosis of Cancer, West China Hospital, Sichuan University, Chengdu, China; ^8^ Breast Cancer Center, Shandong Cancer Hospital and Institute, Jinan, China; ^9^ Breast Tumor Center, Sun Yat-Sen Memorial Hospital, Sun Yat-Sen University, Guangzhou, China; ^10^ 3rd Department of Breast Cancer, Tianjin Medical University Cancer Institute and Hospital, Tianjin, China; ^11^ Henan Breast Cancer Center, Henan Cancer Hospital, Zhengzhou, China; ^12^ Department of Breast Surgery, The Third Affiliated Hospital of Harbin Medical University, Harbin, China; ^13^ Department of Breast Surgery, Xiangya Hospital, Central South University, Changsha, China; ^14^ Department of Breast Surgery, Peking University Third Hospital, Beijing, China; ^15^ Department of Breast Cancer Center, The Fourth Hospital of Hebei Medical University, Shijiazhuang, China

**Keywords:** neoadjuvant chemotherapy, trastuzumab, pathological complete response, clinical trial, carboplatin, anthracycline

## Abstract

This trial was designed to compare the efficacy and safety between epirubicin (E) and carboplatin (C) in combination with paclitaxel (P) and trastuzumab (H) in neoadjuvant setting. In 13 Chinese cancer centers, 100 patients with HER2-positive, locally advanced breast cancer were 1:1 randomized to receive medication as follows: trastuzumab and paclitaxel weekly combined with carboplatin weekly for PCH group, or epirubicin every 3 weeks for PEH group. Patients were given 4 to 6 cycles of chemotherapy. The primary endpoint was pathologic complete response (pCR) rate, which was no significant difference in PCH and PEH regimen (39.1% vs. 48.8%; p=0.365). However, PEH regimen achieved higher pCR in luminal-B (HER2-poitive) subgroup (55.0% vs. 24.0%; p = 0.033), but not in ERBB2+ subgroup (42.9% vs. 57.1%; p = 0.355). PEH regimen showed a favorable efficacy in PIK3CA mutated subgroup (69.2% vs.23.5%, p=0.012). No significant difference was observed in the subgroup analysis of TP53 mutation status, PTEN expression, FCGR2A SNP and FCGR3A SNP. Both regimens as neoadjuvant chemotherapy achieve similar efficacy and safety. PEH might improve pCR rate, especially in the luminal-B subtype and PIK3CA mutation subtype. PEH is feasible and less likely to increase the incidence of acute cardiac events compared to PCH.

## INTRODUCTION

Neoadjuvant therapy has emerged as a successful approach to convert patients that are inoperable at diagnosis to operable, or to make breast conserving surgery possible instead of mastectomy. A pathologic complete response (pCR) after neoadjuvant therapy is a surrogate of excellent outcome of human epidermal growth hormone receptor 2 (HER2) positive breast cancer [1]. Over the past few years, a large number of clinical trials have been conducted in neoadjuvant setting, in an attempt to further refine therapeutic strategies for patients with breast cancer. Such trials have typically aimed at selecting the better regimen for each subtype, optimizing dosage of each reagent, different sequences of similar combinations of reagents, different time intervals of drug administration, and exploring the role of novel reagents.

In a pilot study, anthracycline and paclitaxel combined with trastuzumab showed promising efficacy in patients with metastatic disease, which did not cause any symptomatic cardiac dysfunction [2]. In the pre-trastuzumab era, Stearns et al. showed in a small group of patients with HER2-positive tumors that the likelihood of achieving clinical complete response was higher after anthracyclines than after taxanes treatment [3]. Relatively little attention has been paid to the concurrent combination of anthracyclines and taxanes in trastuzumab-based regimens in neoadjuvant treatment. A few trials examined carboplatin in HER2-positive metastatic breast cancer or in neoadjuvant setting, but the results were controversial [4-7]. Therefore, we designed this clinical trial to compare efficacy and safety between anthracycline and carboplatin, when combined with paclitaxel and trastuzumab. Our primary objective was to compare the pCR rates between the two treatment groups. Secondary endpoints included clinical response and safety analysis. In addition, we did an explorative analysis to identify potentially targetable genetic alterations that are associated with resistance to the regimens investigated in this study.

## RESULTS

### Baseline characteristics

**Figure 1:**
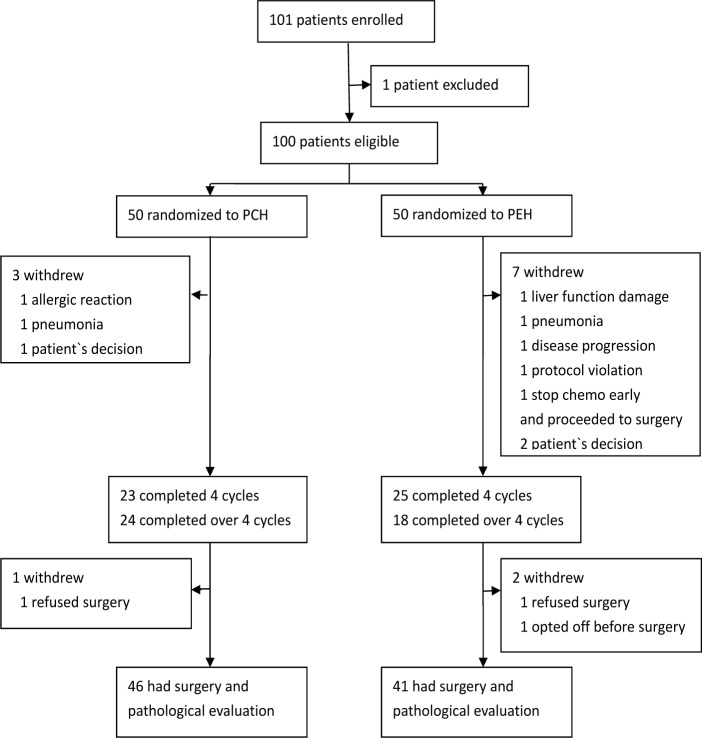
Flow of patients throughout the study

From Aug, 2011 to May, 2012, 101 patients were enrolled from 13 medical centers in China into the clinical trial. Of these patients, 1 patient did not meet inclusion criteria, 50 were randomly assigned to the PCH group and 50 to the PEH group (Figure 1). Baseline patients' characteristics were well balanced between groups (Table 1). Three patients in PCH and 7 patients in PEH group did not complete at least 4 cycles chemotherapy because of AE, disease progression, withdrawal of consent or immediate surgery. After completing at least 4 cycles chemotherapy, 1 patient in PCH and 2 patients in PEH withdrew from this clinical trial. As predefined in the protocol, these patients were excluded from the analysis. In whole cohort, median age was 48 years (range 29-65 years), 61 patients were premenopausal. 49 patients presented with clinical TNM stage II and 51 patients with stage III. 45 patients were ER positive and 37 patients were PR positive. The cut-off value of Ki67 was 14%, 79 patients expressed high level of Ki67. All patients had LVEF over 55% and the median was 66% (range 56.5%-83%).

**Table 1:** Patients characteristics at baseline

	PCH (N=50)	PEH(N=50)	*P* value
Median age, yr(range)	48(29-65)	47.5(30-63)	0.225
Menopausal status			
Premenopausal	30	31	0.544
Postmenopausal	20	16	
Clinical tumor stage			
cT1	1	1	0.970
cT2	32	34	
cT3	13	12	
cT4	4	3	
Clinical lymph node stage			
cN0	7	8	0.511
cN1	21	27	
cN2	15	9	
cN3	6	6	
Estrogen receptor			
Positive	26	19	0.183
Negative	23	29	
Progesterone receptor			
Positive	20	16	0.446
Negative	29	32	
Ki67 index			
≥14%	40	39	0.592
<14%	6	8	
LVEF			
Median, range	67(58-79)	65(56.5-83)	0.443
Chemotherapy cycle			
4 cycles	23	25	0.382
Over 4 cycles	24	18	
FCGR2A			0.278
AA	22	14	
AG/GG	20	21	
FCGR3A			0.298
TT	19	20	
TG/GG	23	15	
PIK3CA status			0.765
Wide type	25	22	
mutated	17	13	
TP53 mutation			0.868
Wide type	22	19	
Mutated	20	16	
PTEN			0.693
Loss	11	9	
Normal	35	35	

### Efficacy and responses

**Figure 2:**
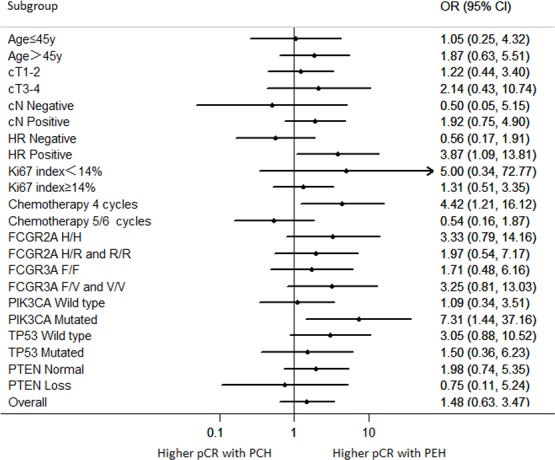
Odds ratios for achieving a pCR according to subgroups

Over 90% of the patients experienced a clinical objective response (CR or PR) assessed by palpation, ultrasonography and/or MRI. After 2 cycles and 4 cycles, the overall clinical response did not differ between two cohorts (Table 2). During the treatment, one patient in PEH group had tumor progress. Two patients in both cohorts underwent breast-conserving surgery (BCS). 18 (39.1%) of 46 patients in the PCH group and 20 (48.8%) of 41 patients in the PEH group achieved pCR (ypT0/is, ypN0; OR 1.48 95%CI 0.63-3.47; *p* = 0.365). No significant difference was noted when other definitions of pCR (ypT0/is, ypN0/+) were used. Based on Miller & Payne grade, no significant difference in tumor regression was observed in both cohorts (*p* = 0.43). Performing subgroup analysis of patients in the PCH group, 13 (54.2%) of 24 patients who received 5/6 cycles of PCH achieved pCR, while 5 (22.7%) of 22 patients who received 4 cycles had pCR (*p* = 0.029). Whereas in PEH group, subgroup analysis revealed no significant difference in pCR rate in patients who were given different number of cycles of PEH (56.5% *vs*. 38.9%, *p* = 0.262). Univariate analysis for clinical characteristics predicting pCR was performed in whole patient population (S.Table 1), revealing no significant predictor for pCR. However, chemotherapy regimen (PCH *vs*. PEH) was found to be an independent factor associated with pCR in multivariate analysis (OR: 3.606, 95%CI: 1.153-11.274, *p* = 0.027). Figure 2 showed the odd ratio (OR) of achieving pCR comparing PCH versus PEH with different characteristics. The results of this analysis revealed that treating patients with hormone receptor (HR) positive breast cancer with PEH regimen had a significantly higher chance to achieve pCR compared with PCH regimen (OR: 3.87, 95%CI: 1.09-13.81, *p* = 0.033). For patients receiving 4 cycles of neoadjuvant chemotherapy, treatment of PEH also significantly increased chance of achieving pCR compared with PCH treatment(OR: 4.42, 95%CI: 1.21-16.12, *p* = 0.021).

**Table 2:** Clinical and pathological evaluation

	PCH group	PEH group	OR [95% CI]	*P* value
ypT0/is,ypN0			1.481 (0.632-3.472)	0.365
No	28	21		
Yes	18	20		
ypT0/is,ypN0/+			1.645 (0.704-3.847)	0.249
No	27	19		
Yes	19	22		
MP grade for breast			NA	0.431
MP 5	19	22		
MP 4	11	8		
MP 3	5	6		
MP 2-0	11	5		
Clinical response after 2 cycles			0.651(0.171-2.474)	0.740
CR	2	3		
PR	40	40		
Overall (CR or PR)	42	43		
SD or PD	6	4		
Clinical response after 4 cycles			3.721(0.399-34.715)	0.366
CR	7	11		
PR	36	29		
Overall (CR or PR)	43	40		
SD or PD	4	1		

Safety and toxicity Most adverse events were of grades 1-2 (S. Table 2). The most frequently occurring grade 3/4 adverse events were neutropenia and granulocytopenia. The incidence of febrile neutropenia was 8% in PCH group and 14% in PEH group. PEH regimen caused higher frequency of grade 3/4 leucopenia than PCH regimen (26% *vs* 8%, *p* = 0.017). Nausea, vomiting and fatigue were the most frequent non-hematological toxic effects, but these cases were mostly mild or moderate. Symptomatic congestive cardiac failure was not observed. 4 patients in PCH group and 5 patients in PEH group were admitted to hospital for treatment-related toxicities. No treatment-related death occurred.

S. Figure 2 showed LVEF at baseline, and during neoadjuvant treatment after 2 and 4 cycles. All patients in both groups maintained normal LVEF throughout the study. After 2 cycles, over 10% reduction of LVEF was observed in 5 patients (11.9%) in PCH group and 3 patients (7.7%) in PEH group, while it was 3 patients (7.3%) in PCH group and 1 patient (2.7%) in PEH group had over 10% LVEF reduction after 4 cycles. No patient had LVEF decrease to less than 50% at any time during the study. None of the patients receiving chemotherapy developed cardiac failure and there were no chemotherapy discontinuation due to cardiac toxicity.

### Biomarker analysis

*PIK3CA* mutation was detected In 30 (39.0%) out of 77 tumor samples available, which were balanced between HR-positive and HR-negative tumors (46.3% *vs*. 30.5%, *p* = 0.156). The presence of *PIK3CA* mutation was not significantly associated with lower pCR rate. 43.3% of patients with PIK3CA-mutant tumors achieved pCR compared with 48.8% in the wild-type subgroup (OR: 0.80, 95% CI: 0.316 to 2.024; *p* = 0.637) (Supplementary Table 1). Based on *PI3KCA* mutation status analysis, there is no statistically significant difference in efficacy to *PIK3CA* wide type tumors between PEH and PCH group (*p* = 0.884). However, PEH regimen showed a favorable efficacy in patients with *PIK3CA* mutation (69.2% *vs*.23.5%, *p* = 0.012).

In this data set, the frequency of *FCGR2A* polymorphism at amino-acid 131 was 36 H/H (46.8 %), 36 H/R (46.8 %), and 5R/R (6.4 %). The frequency of *FCGR3A* polymorphism at amino-acid 158 was 39 F/F (50.6 %), 37 F/V (48.5 %), and 1 V/V (1.3 %). The two gene SNPs were not correlated with pCR rate. Distribution of the *HER2* mutation (A763T and T862A) was 2 (2.6 %), both of which did not achieved pCR. *TP53* mutations in exon 4, 5, 6, 7, 8 were detected in 32 (41.6%) patients, while 20 (22.2%) patients had PTEN loss. These potential biomarkers were not statistically significantly associated with pCR in the whole cohort and subgroup analyses.

## DISCUSSION

In this clinical trial, there was no significant difference on pCR rate between two groups. In HR+ subgroup and PIK3CA mutation subgroup, the PEH regimen could bring higher therapeutic benefit. Within the HER2-positive population, chances of achieving pCR was more common for patients with HR negative tumor than with HR positive tumor [8, 10, 11]. Our results showed that PEH improved the pCR rate in HR positive subtype. Few studies have evaluated concurrent administration of anthracycline-based chemotherapy and trastuzumab in neoadjuvant setting [7, 12, 13]. In contrast to previous reports, anthracycline followed by taxanes with trastuzumab-based neoadjuvant treatment was both effective and well tolerated [14, 15].

The biological rationale for our conclusions was based on limited preclinical and clinical observations. ER negative subtype is sensitive for chemotherapy and trastuzumab, different combinations of agents may achieve similar high pCR rate. Active agents and different regimen may improve the efficacy in luminal-B (HER2-positive) subtype [6, 8, 9, 12]. Results of the GeparSixto study showed an increase of the pCR rate from 37.2% to 46.7% by the addition of carboplatin. An absolute increase by > 20% was observed in patients with TNBC (37.9% *vs*. 58.7%), but no increase in patients with HER2-positive breast cancer (36.3% *vs*. 33.1%) [7]. It is possible that carboplatin and docetaxel-induced senescence may render cells resistant to further therapies [16, 17]. In a retrospective study of 300 patients, PH-FECH (paclitaxel and trastuzumab and FEC75) shows a higher pCR rate and recurrence-free survival (RFS) advantage than TCH (docetaxel, carboplatin and trastuzumab) [13]. Previous work has shown that activation of the HER family of receptors is associated with up regulation of Topo-IIα and increase in sensitivity to doxorubicin [18]. 30% of HER2-potitive breast cancer express p95HER2, which is resistant to trastuzumab. However doxorubicin sensitized p95HER2 to trastuzumab in patient-derived xenografts [19]. Therefore, it is conceivable that, in HR positive and HER2 positive subgroups, which are less responsive to chemotherapy and targeted therapy, concurrently adding anthracycline may lead to additional benefit in our study.

The treatment benefits need to be weighed against the risk of cardiotoxicity. In a pivotal phase III trial in metastatic breast cancer, the concurrent administration of anthracyclines and trastuzumab resulted in an unacceptably high rate (27%) of cardiotoxicity [20]. However, emerging evidence have showed that concurrent use of trastuzumab and anthracycline may not be associated with LVEF dysfunction. When trastuzumab was given concurrently with paclitaxel after completion of doxorubicin in adjuvant setting, the incidence of symptomatic cardiac failure was 2.8%-4.1% [21, 22]. In the monitoring of cardiac function under strict application, low cumulative dosage of anthracycline combined with trastuzumab may cause low occurrence of cardiac function failure. Despite concurrent use of doxorubicin, paclitaxel and trastuzumab in the NOAH trial, incidence of left ventricular dysfunction was less than 2%. In the GeparQuattro trial, only one patient treated with concurrent administration of trastuzumab with epirubicin reported persistent decrease in LVEF to less than 50% [23]. Additionally, the concurrent group in Z1041 trial showed that concurrent use of trastuzumab and anthracycline was not associated with an increased risk of cardiac dysfunction [12]. Consistently, no patient had LVEF decrease to less than 50% at any time during our study. Our results support the notion that trastuzumab can be given concurrently with anthracycline with low cardiac toxicity.

PIK3CA mutation in HER2-positive tumors, leading to PI3K pathway irregulation, has been shown to be important in developing resistance to trastuzumab [24]. PIK3CA mutation has been reported in approximately 20% to 30% of HER2-positive tumors by sanger sequencing of mutation hot spots [25-27]. In this study, next generation sequencing was used to detect mutations in exon4, 9, 20 of PIK3CA with detection sensitivity of 1% mutated cells. In the HER2-positive population enrolled in this study, our results showed that HR positive and HR negative tumors had similar PIK3CA mutation frequencies, which is consistent to previous pooled analysis [25]. In the neoadjuvant GepaSixto study, the presence of PIK3CA mutation was significantly associated with lower pCR rate, especially in the HR positive population. However, in the Geparquattro and GeparQuinto studies using anti-HER2 therapy, pCR rate was not significantly associated with PIK3CA mutation and HR status, which agreed with our results [25]. A retrospective study suggested that PIK3CA mutation status may be a predictor for clinical response of the combination regimen of epirubicin and docetaxel in neoadjuvant setting [28]. Comparing PCH with PEH in this study, our results indicated that PIK3CA mutation may be a potential predictor of clinical outcome for PEH regimen.

The TP53 gene is a prime candidate for predicting the response of tumors to classic chemotherapy, however other trials did not confirmed the conclusion [29]. In other studies, the TP53 mutation rate was between 20-50% in HER2 overexpressing subtype, which is similar to our results [27, 30]. Previous retrospective studies investigating the correlation of FCGR3A/2A genotypes with clinical outcome to trastuzumab-based therapy yielded discordant results [31-33]. Our study did not show a correlation between FCGR3A-V/F and FCGR2A-H/R SNPs and pCR in patients treated with trastuzumab. While preclinical studies have shown that PTEN loss may contribute to trastuzumab resistance, previous clinical studies have failed to give us a clear answer [34-36].

From the results of this study, both PCH and PEH are suitable options for patients with locally advanced, HER2-positive breast cancer. Results of exploratory analysis implied that HR+ and PIK3CA mutation might be a positive predictor for PEH treatment outcome. Our results confirmed earlier findings that concurrent administration of trastuzumab and anthracycline is not associated with an increased risk of cardiac dysfunction. However, our study may be limited by its small sample size, which is underpowered for the subgroup analysis. Although trastuzumab combined with chemotherapy remains the standard of care, dual HER2-blockade strategies have significantly improve pCR rate [8, 37]. The final survival results of the neoadjuvant trials using dual HER2 inhibition may help elucidate the final role of this approach for early stage disease. It remains to be determined whether the differences in outcomes between the groups in this trial were the result of the antitumor effect of anthracycline, or synergy from the combination of anthracyclines, paclitaxel with trastuzumab. Understanding the mechanism that underlies sensitivity and resistance to various reagents will help investigators develop rational combinations and sequences of drugs to further improve clinical outcome [38]. Future randomized studies with larger prospective cohorts and longer term follow-up are needed to validate these findings.

## PATIENTS AND METHODS

### Study design

This is a multicenter, randomized, phase II, parallel-group trial conducted in 13 leading cancer centers in China. Untreated patients with histologically confirmed stage II-III, HER2-positive breast cancer were considered eligible. Other inclusion criteria included the following: age between 18–70 years; infiltrating primary breast cancer with the longest clinical diameter of more than 3.0 cm; assessable tumor in the breast without evidence of distant metastasis measured by breast mammogram, magnetic resonance imaging, chest computed tomography scan, abdominal ultrasound and bone scan; Eastern Cooperative Oncology Group performance status of 0-1; left ventricular ejection fraction (LVEF) > 55%. Patients were required to have an adequate hematopoietic function (absolute neutrophil count≧1.5×10^9^/L, platelet count≧100×10^9^/L, and hemoglobin level≧100g/L), adequate hepatic and renal function (Serum total bilirubin and Serum creatinine ,<1.5×ULN (upper limit of normal), aspartate aminotransferase and alanine aminotransferase<2.5×ULN). All patients provided written informed consent before study enrollment. The protocol was reviewed by all responsible local ethics committees. This trial is registered in ClinicalTrials.gov, with identifier NCT01428414.

### Procedures

Randomization was done centrally at the operation office with sequentially numbered, opaque, sealed envelopes. The treatment plan is illustrated in S. Figure 1. Trastuzumab (4 mg/kg loading dose followed by 2 mg/kg) and paclitaxel (75 mg/m^2^) weekly combined with carboplatin (AUC = 2) weekly for PCH group or epirubicin (75 mg/m^2^) every 3 weeks for PEH group. Patients were given at least 4 cycles but no more than 6 cycles under discretion of physicians, which were common accepted cycles in concurrent neoadjuvant regimens [8, 9]. Breast surgery with axillary dissection or breast conserving surgery was performed 2–4 weeks after the last chemotherapy dose. Surgery type was decided at the surgeon's discretion. Chemotherapy regimen was at the doctors' discretion and began within 4 weeks postoperatively. One year of trastuzumab in total was recommended for all patients. Patients with estrogen or progesterone receptor-positive disease received endocrine treatment based on National Comprehensive Cancer Network (NCCN) guideline.

### Assessment

Immunohistochemistry (IHC) assessment of estrogen receptor (ER), progesterone receptor (PR), HER2 expression, Ki67, PTEN was conducted in paraffin-embedded tumor samples biopsied before neoadjuvant treatment according to the guidelines from the American Society of Clinical Oncology and the College of American Pathologists. HER2 positivity was determined by IHC 3+ or fluorescence in situ hybridization (FISH) positive status. The clinical response in the breast was assessed using a breast MRI, mammogram or ultrasound after every two cycles of neoadjuvant chemotherapy and categorized as a clinical complete response, partial response, stable disease, and progressive disease according to the Response Evaluation Criteria in Solid Tumors Version 1.1. pCR in the breast was defined as the disappearance of residual invasive disease (residual ductal carcinoma in situ allowed) by pathologic examination, and pCR in the axilla was assessed as the absence of positive lymph nodes by hematoxylin and eosin staining. The independent pathological review committee included 4 senior pathologists, who did not belong to the 13 participating medical centers. The primary endpoint of this study was pCR in the breast and axilla. Toxicity was evaluated at every cycle and recorded according to the Common Terminology Criteria for Adverse Events (CTCAE) version 4.0.

Gene mutations were evaluated in tumor samples from FFPE core biopsies taken before therapy with a tumor content more than 50% determined by using next generation deep Ion DNA AmpliSeq^TM^ (Average depth of coverage = 1000×, Life Technologies). Hot-spot mutations were detected in whole exon 4^th^, 9^th^ and 20^th^ of *PI3KCA*, exon 5^th^-8^th^ exon of *TP53*, exon 19^th^-25^th^exon of *HER2*. DNA was purified from whole blood samples using QIAamp DNA Blood Mini Kit (QIAGEN, CA), and detected by PCR amplification and classical Sanger sequencing of regions containing the *FCGR3A* 158 V/F and *FCGR2A* 131 H/R SNPs.

### Statistical analysis

Patients' tumor characteristics and adverse events were summarized by descriptive statistics. Treatment groups were compared by continuity corrected two-sided Pearson's χ2 test or Fisher's exact test where appropriate. The response rates and odds ratios (ORs) with 95% confidence intervals (CIs) were calculated. Univariate logistic regression was used in subgroup analyses, while interaction effects were also displayed using forest plots. A multivariable logistic regression was used to adjust for the baseline factors. All P values were two-sided and a P value of less than 0.05 was considered statistically significant. Statistical analysis was performed using SPSS v.12.0 and STATA v.11.0.

## SUPPLEMENTARY FIGURES AND TABLES


