# Supplementary material for: Efficacy and safety analysis of trastuzumab and paclitaxel based regimen plus carboplatin or epirubicin as neoadjuvant therapy for clinical stage II-III, HER2-positive breast cancer patients: a phase 2, open-label, multicenter, randomized trial
Source: Oncotarget. 2015 Jun 2;6(21):18683–92. doi: 10.18632/oncotarget.4337 (PMC4621920; doi:10.18632/oncotarget.4337)
Supplement: Supplementary file 1 [file oncotarget-06-18683-s001.pdf]

Efficacy and safety analysis of trastuzumab and paclitaxel based regimen plus carboplatin or epirubicin as neoadjuvant therapy for clinical stage II-III, HER2-positive breast cancer patients: a phase 2, open-label, multicenter, randomized trial

Supplementary Material

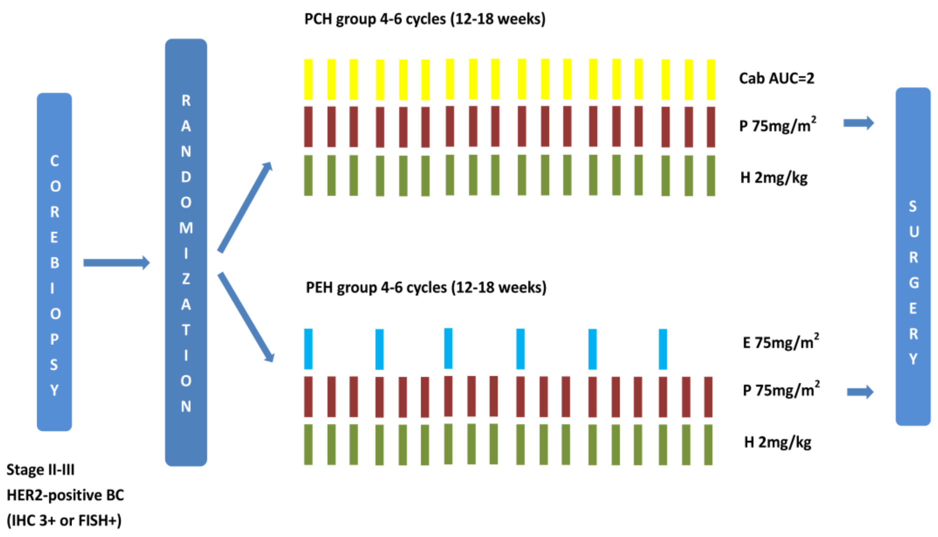

S.Figure 1: Treatment plan

Abbreviations: AUC, area under the concentration-time curve; BC, breast cancer; Car, carboplatin; FISH, fluorescence in situ hybridization; H, trastuzumab; IHC, immunohistochemistry; P, paclitaxel.

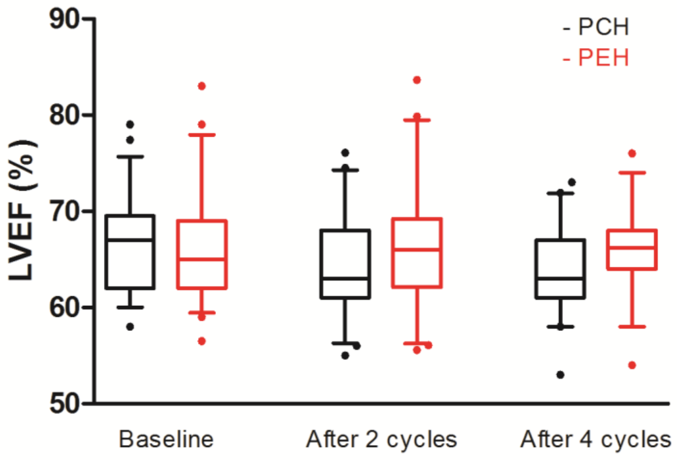

S.Figure 2: Left ventricular ejection fraction (LVEF) per treatment arm.

Central horizontal lines show medians, limits of shaded boxes show 50th percentile, horizontal lines outside

S.Table 1: Odds ratios for pathological complete response according to subgroups

		Univariable		Mutivarible	
	**Category**	**Odds ratio (95% CI)**	***P* value**	**Odds ratio (95% CI)**	***P* value**
**Arm**	PCH vs PEH	1.481 (0.632-3.472)	0.365	3.606 (1.153-11.274)	0.027
**Age**	＜45y vs ⩾45y	1.218 (0.504-2.945)	0.662	1.053 (0.338-3.279)	0.930
**cT**	cT1-2 vs cT3-4	0.490 (0.19q-1.258)	0.135	0.407 (0.134-1.232)	0.112
**cN**	Negative vs positive	0.721 (0.212-2.447)	0.599	0.332 (0.054-2.057)	0.236
**Ki67**	＜14% vs ⩾14%	1.737 (0.479-6.295)	0.397	2.483 (0.407-15.153)	0.324
**HR status**	Negative vs positive	0.607 (0.259-1.426)	0.251	0.550 (0.177-1.711)	0.302
**Chemotherapy cycles**	4 cycles vs 5-6 cycles	1.364 (0.583-3.190)	0.474	2.671 (0.881-8.794)	0.106
**FCGR2A**	H/H vs H/R and R/R	0.561 (0.224-1.406)	0.216	0.330 (0.098-1.110)	0.073
**FCGR3A**	F/F vs F/V and V/V	1.294 (0.521-3.214)	0.578	1.348 (0.432-4.209)	0.607
**PIK3CA**	Wid type vs mutated	0.799 (0.316-2.024)	0.637	0.799 (0.250-2.550)	0.705
**TP53**	Wid type vs mutated	1.404 (0.558-3.533)	0.471	0.813 (0.235-2.808)	0.743
**PTEN**	Normal vs Loss	1.103 (0.378-3.219)	0.857	0.694 (0.159-3.024)	0.627

S.Table 2: Cardiac and most common adverse events reported as possibly, probably, or definitely related to treatment

	HPC group (*N*=50)		HPE goup (*N*=50)	
**Adverse event**	**Grade 1/2**	**Grade 3/4**	**Grade 1/2**	**Grade 3/4**
**Anemia**	30	7	33	5
**Febrile neutropenia**	0	4	0	7
**Neutropenia**	17	28	12	35
**Leucopenia**	14	4	15	13
**Thrombocytopenia**	10	3	5	1
**Granulocytopenia**	22	23	20	26
**ALP increased**	3	0	1	0
**Bilirubin increased**	26	1	23	2
**AST increased**	16	1	13	0
**ALT increased**	22	4	22	0
**Hand-foot syndrome**	3	0	9	0
**Diarrhea**	8	1	6	1
**Nausea**	29	0	31	0
**Vomiting**	23	0	21	0
**Fatigue**	24	0	22	1
**Peripheral neuropathy**	6	0	7	0
**Arthralgia/myalgia**	0	0	0	0
**Congestive heart failure**	0	0	0	0
**Palpitation**	4	0	5	0
**Lung infection**	1	1	1	1
